# Strategies for successful designing of immunocontraceptive vaccines and recent updates in vaccine development against sexually transmitted infections - A review

**DOI:** 10.1016/j.sjbs.2022.01.006

**Published:** 2022-01-07

**Authors:** A.S. Vickram, Kuldeep Dhama, S. Thanigaivel, Sandip Chakraborty, K. Anbarasu, Nibedita Dey, Rohini Karunakaran

**Affiliations:** a Department of Biotechnology, Saveetha School of Engineering, Saveetha Institute of Medical and Technical Sciences, Tamil Nadu, India; b Division of Pathology, ICAR-Indian Veterinary Research Institute, Izatnagar, Bareilly, Uttar Pradesh 243122, India; c Department of Veterinary Microbiology, College of Veterinary Sciences &amp, Animal Husbandry, R.K.Nagar, West Tripura, Pin- 799008, India; d Department of Bioinformatics, Saveetha School of Engineering, Saveetha Institute of Medical and Technical Sciences, Tamil Nadu, India; e Unit of Biochemistry, Faculty of Medicine, AIMST University, Semeling, Bedong, Kedah, Malaysia

**Keywords:** Vaccine, Immunocontraception, Sexually transmitted infections, Herd immunity, Syphilis, Herpes simplex virus, Antigens, Immunogens

## Abstract

**Background:**

The world population is continuously growing. It has been estimated that half of the world’s population is from the Asian continent, mainly from China and India. Overpopulation may lead to many societal problems as well as to changes in the habitat. Birth control measures are thus needed to control this growth. However, for the last 50–60 years, there have not been any improvements in the field of contraception. Nevertheless, the immunocontraceptive vaccine is an emerging field, and it might be the only replacement for the existing mode of contraception for the next millennium. Sexually transmitted infections (STIs) are frequent, and their transmission rate increases yearly. As antibiotics are the prevailing treatment for this kind of infections, resistance in humans has increased; therefore, having effective antibiotic treatments for STIs is now a concern. Vaccines against STIs are now needed. It is thought that the improvements in the fields of proteomics, immunomics, metabolomics, and other omics will help in the successful development of vaccines.

**Objective:**

To collect and review the literature about recent advancements in immunocontraception and vaccines against sexually transmitted diseases/infections.

**Methods:**

Reliable scientific databases, such as PubMed Central, PubMed, Scopus, Science Direct, and Goggle Scholar, were consulted. Publications bearing important information on targeted antigens/immunogens for contraceptive vaccine design and advancements in vaccine development for STIs were gathered and tabulated, and details were analyzed as per the theme of each study.

**Results:**

Important antigens that have a specific role in fertility have been studied extensively for their contraceptive nature. Additionally, the advancements in the screening for the best antigens, according to their antigenic nature and how they elicit immune responses for an extended period were also studied. Herd immunity for STIs and advancements in the development of vaccines for syphilis, gonorrhea, and herpes simplex virus were also studied and tabulated in this review. An extensive knowledge on STIs vaccines was gained.

**Conclusion:**

This extensive review is aimed to provide insights for active researchers in vaccinology, immunology, and reproductive biology. Advancements in the development of vaccines for different STIs can be gathered as a wholesome report.

## 1 Introduction

**Fig. 1:**
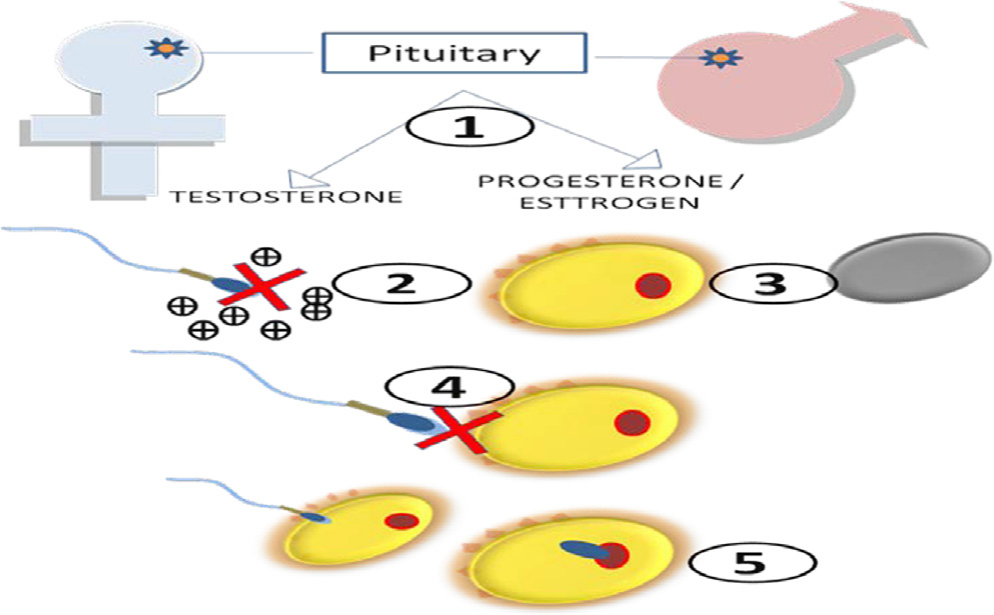
**Strategies for contraceptive vaccine design.** 1. Disturbing the hormone axis leading to inappropriate secretions results in contraception. 2. Sperm cell needs to bind to prostasomes for motility and establishing fertility. Target antigens can prevent this binding and works as contraception. 3. Targeting immunogenic proteins responsible for an ovum may result in anovulation. 4. Thus, an essential step in the fertilisation process- binding sperm cell with egg and its further fusion can be prevented, and contraception is achieved. 5. A large number of immunogenic proteins are involved in suppressing maternal response to sperm cell and embryo. Targeting any of those proteins can successfully prevent implantation of the fetus, so pregnancy can be avoided.

The world population is growing at an alarming rate each year (Moore et al., 2016), and this growth rate in developing countries is two times the rate in developed nations (Uniyal et al., 2016). Half of the world’s population is contributed by Asian countries, specifically by India and China. Besides, contraceptive usage and research are in their infancy till 1960 (Pohjoranta et al., 2020). This leads to 46 million abortions each year in the developing nations (Ganatra et al., 2017), which are also due to a lack of proper temporary contraceptive practices (Dahal et al., 2017 2(1):1005). Government agencies only promote condom usage for men as an effective contraception method in the developing nations (Kidan and Azeze, 2017, Girma et al., 2017). But condom usage is not 100% effective for birth control (Magnani et al., 2007). Once again, this lack of effectivity leads to abortions and further consequences that must be faced by women in the developing countries (Barroso and Babanto, 2016). Birth control methods available in developed nations include birth control implants (Cromer et al., 1994), patches (Hampton, 2016, Kuehn, 2008), pills (Delavande, 2008), birth control shots (Jayaraman, 2018), sponges (for vaginal sex) (Reed, 2014), vaginal rings (Murphy et al., 2016), breastfeeding as an alternate type of birth control (Houvèssou et al., 2020), cervical caps (Zapata et al., 2015), condoms (Clark et al., 2014), diaphragms (Beksinska, 2016), female condoms (Wiyeh et al., 2020), fertility awareness programs (Malarcher et al., 2016), intrauterine devices (IUDs) (Lopez et al., 2015), outercourse and abstinence (Bakaroudis, 2014), spermicides (Susmiarsih et al., 2017), and vasectomy (Mandell et al., 2021). However, developing nations are widely using condoms as sole contraceptives, which are only 70% effective for birth control. Countries like India and China are strongly recommending controlling overpopulation given that food and clean water supplies are limited and that fulfilling people’s basic needs often leads to habitat and environmental destruction (Edet). Therefore, modern or novel contraception methods with a 100% success rate for birth control are still needed; they would reduce unnecessary abortions, protecting women’s health (Hanna et al., 2016). Although cost affordable, effective methods of contraception are available in the market in both developed and developing countries, pharmaceutical companies are still investing much money in search for new contraceptive methods (Rubin, 2017). Moreover, existing methods are still not affordable for people in rural areas of countries like India and China, and governments should focus in reducing the unmet demand. For the last 30 or 40 years, numerous reproductive biologists and immunologists have been trying to develop a contraceptive vaccine with a 100% success rate in birth control. Immunocontraception might be the novel contraceptive method for the next millennium (Talwar et al., 2015. Fig. 1, Tables 1 and 2.

**Table 1:** Important and explored antigens/immunogens for contraception.

**Targeted Immunogens/Antigens**	**Species**	**Mechanism**	**Author studied and year**
Porcine zona pellucida (pZPC)	Wallaby White-tailed deer, Wild horses	It stops the attachment of sperm with egg and thus the fertilization	Curtis et al., 2002, Curtis et al., 2002, Turner et al., 2002, Turner et al., 2002, Shideler et al., 2002, Shideler et al., 2002, Kirkpatrick et al., 1996 50, (Kirkpatrick et al., 1996 50) (Tumova et al., 2021)
Virally vectored zona pellucida	Rabbit vertebrate Pests Mouse	Produces Follicular damage after immunisation and thus prevent the meeting of sperm and egg for fertilization	Hardy et al., 2006, Hardy et al., 2006, Lloyd et al., 2003, Lloyd et al., 2003, Hardy, 2007, Hardy, 2007, Redwood et al., 2008 (Redwood et al., 2008) McLeod et al., 2008, McLeod et al., 2008
Gonadotropin-releasing hormone (GnRH)	Swine Mouse Horse White-Tailed Deer	Stops or lessens the production of LH and FSH hormone, which helps in controlling the gamete production and also the secondary sexual characteristics in both male and female	Killian et al., 2006, Killian et al., 2006, Miller et al., 2008, Miller et al., 2008, Han et al., 2016, Han et al., 2016, Goodwin et al., 2015, Goodwin et al., 2015, Barranco et al., 2015, Barranco et al., 2015 (Madbouly et al., 2021) Pinna et al., 2015, Pinna et al., 2015
SP-17	Domestic kittens mice	Stops the sperm capacitation and sperm motility, steps adherence of prostasomes and sperm cell which is much needed for fertilisation	Minhas et al., 2016, Minhas et al., 2016, Kaur and Prabha, 2014, Kaur and Prabha, 2014, Lea et al., 1997, Lea et al., 1997
Rodent epididymal protein, DE, belongs to cysteine-rich secretory protein (CRISP)	Rodents	It prevents the sperm and egg fusion	Da Ros et al., 2015, Da Ros et al., 2015, Yu et al., 2015, Yu et al., 2015, Ellerman et al., 1998, Ellerman et al., 1998
Eppin- Male reproductive traced antigen	Mice Monkey	Stops sperm motility and engages in entrapment of sperm cell even after liquefaction	Wang et al., 2007, Wang et al., 2007 (O'rand et al., 2006) O'rand et al., 2004, O'rand et al., 2004, O'Rand et al., 2009, O'Rand et al., 2009, Michael et al., 2016, Michael et al., 2016 (Nieschlag)
Bovine bone morphogenetic protein 15 (BMP15)	Mice	Inhibition of normal ovarian follicular development and also anovulation	Persani et al., 2014, Persani et al., 2014, Otsuka et al., 2011, Otsuka et al., 2011

**Table 2:** Importance of Vaccine development for various sexually transmitted infections.

**Sexually transmitted diseases**	**Vaccine development strategies done**	**The key factor that needs to be addressed**	**The author studied and year**
Syphilis	Gaps in current biomedical knowledge and technologies for syphilis treatment should be focused on. The development of a vaccine for syphilis should be very safe and effective against transmission between sexually active population as well as it should protect against complications including congenital and neurosyphilis	Epidemiologic data and implications on infection and disease for syphilis	Kersh and Lukehart, 2018, Sood et al., 2017, Ambrose, 2016, Kersh and Lukehart, 2018, Gottlieb et al., 2016, Gottlieb et al., 2016, Lithgow et al., 2017, Ambrose, 2016
Gonorrhoea	New vaccine approaches need gonococcal subversion of immune responses, antigenic variations, and gender pose difference should be the best option for effective vaccine design for Gonorrhea, key knowledge about the epidemiology of the gonococcal subversion, advancing basic knowledge to translational research for an effective vaccine.	Pitiable natural immunity, antigen variations, the evolution of gonococcal subversion are the key challenges that need to be addressed for an effective vaccine	Jerse et al., 2014, Lithgow et al., 2017, Zielke et al., 2016, Jerse et al., 2014, Ratto-Kim et al., 2018, Wetzler et al., 2016 (Zielke et al., 2016) Wetzler et al., 2016, Russell, 2018
HSV	The unique nature of HSV infection, need to analyse the biological feasibility for vaccine development, Prime-pull strategy as an effective vaccine development for HSV, the evolution of HSV 1 and 2 subversions are found to be the essential things that need to be focused on for the development of a vaccine for HSV 1 and 2.	Protective genital mucosal immunity, focus on preventive vaccine rather than therapeutic vaccine	Dubensky et al., 2018, Wetzler et al., 2016, Dubensky et al., 2017, Dubensky et al., 2018 (Dubensky et al., 2017) (Johnston et al., 2016) Hensel et al., 2017, Long et al., Dooling et al., 2018, Hensel et al., 2017
HPV	The difference between precancerous lesions and HPV led cancer is different to understand, focusing on a quadrivalent vaccine for HPV will be an important move for the HPV vaccine, arranging of large sexual education programs in the young girls will also promote the vaccine, the development and advancement in DNA type vaccine for HPV will be another focus	Obtaining recovered epidemiologic information on infection and disease worldwide and facilitating clinical evaluation and vaccine introduction in male and female will be the key challenge	Brekke et al., 2018, Dooling et al., 2018, Di Mario et al., 2015, Brekke et al., 2018, Hsieh et al., 2015, Di Mario et al., 2015, Schiller, 2016, Hsieh et al., 2015, Agarwal et al., 2018, Schiller, 2016 (Agarwal et al., 2018) Best & Pai, 2015 (Oh et al)

The development of contraceptive vaccines is the core work for many immunologists. Still, the anti-human chorionic gonadotrophin (hCG) vaccine is the only immunocontraceptive vaccine that might enter clinical trials (Talwar et al., 2015). For better vaccine development, the target antigen should be competitive and good enough for controlling human fertility. Besides, the vaccine should be completely antigenic and elicit long-lasting immunogenic responses in the female or male bodies to be infertile. The vaccine should also be affordable, be very safe for human use, compete with already existing techniques with a near or 100% success rate for birth control, be reversible, and meet the unmet demand for contraception (Samoylova et al., 2017). All the above-mentioned modes of contraception may prevent unnecessary abortions or pregnancies, but they do not prevent the spread of sexually transmitted infections (STIs) (Steiner et al., 2016). Therefore, designing a suitable vaccine against STIs is in need as a preventive measure. The number of studies aiming to develop vaccines against STIs is rapidly growing; however, slow progress and a lot of disappointments have been faced (Brotman et al., 2014). There is a lack of understanding regarding STI causing organisms and the transmission mechanisms during sexual intercourse. The development of vaccines for herpes simplex virus (HSV), human papillomavirus (HPV), and Hepatitis B found success even in human trials. However, we have not been able to design successful vaccines for syphilis, *Neisseria*, or HIV, for example (Almeida and Borges, 2015). Until a vaccine for these infections is successful, they would remain transmissible to healthy individuals who are sexually active and have multiple sexual partners. Therefore, preventive rather than therapeutic vaccines are needed (Strauss and Madan, 2016). Nearly three decades ago, the vaccine for hepatitis B was developed. However, the prevalence of this disease is still high in rural areas, especially among adolescents who are highly active in sexual intercourse. This proves that, even after identifying and developing a vaccine for an STI, the governments have to take the necessary steps to ensure the availability of such vaccines in rural areas; they should also conduct awareness programs about STIs and immunization. Accordingly, there is a need for both novel contraceptive vaccines for temporal birth control and for STIs for adolescents and preadolescents. This review emphasizes on significant targeted antigens for immunocontraception, crucial strategies for screening and identifying antigenic targets, STIs, and advancements in vaccine development for various STIs.

## 2 Target antigens in sperm and male reproductive tract for human contraception use

It has been very long believed that semen, especially sperm cells, are highly immunogenic in both genders. In 1932, Baskin and colleagues worked with immunizing women with whole semen from their partners for a prescribed time to check whether it would lead to infertility over a prolonged period (Dominiak et al., 2016). After this, many researchers started working with human semen as a contraceptive; the work on immunocontraception started blooming. Almost 7% of infertile men and 75% of vasectomized men have anti-sperm antibodies in their sera, confirming that there are many chances of developing immunocontraception for human use based on whole semen, defined sperm cells, and/or sperm-specific antigens (Hull; M.G., C.M. Glazener; N.J. Kelly; D.L. Conway; P.A. Foster and R.A. Hinton, , 1985). Nowadays, many sperm antigens (proteins) have been identified as useful for developing immunocontraceptive vaccines. Among these antigens, SP17 was an important antigen in which many researchers worked to elucidate its immunocontraceptive properties (Lea et al., 1997, Mortazavi et al., 2021, Diekman and Herr, 1997, O'Donnell et al., 2021, Mirandola et al., 2015). SP10 (Mortazavi et al., 2021) and SP56 (Bleil and Wassarman, 1990) have also been studied for their role and use in immunocontraception (Frayne and Hall, 1999, Jalalvandi et al., 2021, Naz, 2005, Kerr et al., 1998, Sharma et al., 2017). Other antigens like tNASP (nuclear autoantigenic sperm protein) (Wang et al., 2009, Nagatomo et al., 2016), FA-1 (Naz and Wolf, 1994), SOB-2 (Lefevre et al., 1997, Hay et al., 2014), SPAM1 (McLaughlin et al., 2003, Tecle and Gagneux, 2015), and sperm associated antigen 9 (Jagadish et al., 2006, Lou et al., 2016) have also shown promising results for immunocontraception. These antigens have some spermicidal activity, which also inhibits sperm cell-egg interactions (Archana et al., 2021). This inhibitory effect leads to simultaneous infertility in the female partner when used as immunocontraception or immunotherapy (Shetty et al., 2017).

Izumo, a sperm plasma membrane protein, proved to have many molecular-based functions in the fusion of sperm cell and egg. Izumo protein is also a particularly good target antigen that may act as an immunocontraceptive vaccine for women after immunization (Ellerman et al., 2009). The immunizing Izumo antigen exhibits inhibition of sperm-egg interactions in mouse models. Inoue *et al.* studied whether human Izumo was also involved in sperm and egg fusion (Inoue et al., 2005, Naz, 2014). They observed that sperm and egg fusion mechanisms were significantly impaired. They have also shown that this phenomenon is species specific given that it occurs between the sperm and the zona pellucida (Rubinstein et al., 2006). Inoue *et al.* produced an anti-human-Izumo (a polyclonal antibody),mixed it with a sperm and egg mixture, and incubated it for 45 min. This resulted in no fusion between the sperm and egg because of Izumo inhibition (Naz, 2014). However, if sperm is treated with a standard IgG, the fusion happens. On average, six sperms were able to bind an egg in experiments *in vitro* (Inoue et al., 2005). CD46, DE, and SAMP32 (Hao et al., 2002) are other antigens found on the sperm surface plasma membrane that are not much significant in sperm cell and egg fusion. Out of all antigens on the sperm cell plasma membrane that have been tested, Izumo is the only one found to have a significant role in fusion. Additionally, it can be used as immunocontraception, as evidence by various *in vitro* and *in vivo* studies (Wang et al., 2008, Wang et al., 2009). An *et al.* studied the recombinant plasmid pCXN2-mIzumo as an immunocontraceptive vaccine. *In vitro* fertilization was found to be around 11.07% in mice immunized with pCXN2-mIzumo, whereas it was 36% in the control group (An et al., 2009). This study concluded that pCXN2-mIzumo would be a candidate target for designing a proper immunocontraceptive vaccine (An et al., 2009).

Various studies done in a variety of animal models have reported many potential sperm antigens. However, no single sperm antigen has been authorized for use as a human vaccine since they have their limitations (Tumova et al., 2021). Therefore, researchers are now working with multi antigens to combat the single-antigen effect and to produce a proper immunocontraception method (Ferro and Garside, 2011, Hardy et al., 2008, Naz, 2011). Hardy *et al.* used a multi-antigen single recombinant polypeptide bearing SP56 and ZP1, 2, and 3 to immunize female mice. This resulted in a reduced fertility rate when compared to the control group. In addition, Naz and Aleem combined six different sperm-specific antigens into a single recombinant polypeptide. This recombinant protein, when administrated, was proved to be successful in controlling the fertility rate. Another study utilized acrosomal proteins as multi antigens and succeeded in reducing the fertility rate in monkeys (Kurth et al., 2008).

## 3 Screening novel antigens for effective contraception

It has been observed that, approximately, 5% to 10% of human male infertility is due to anti-sperm antibodies in the sera (Marconi and Weidner, 2017), and that 70% of the vasectomized men also have anti-sperm antibodies (Brubaker et al., 2018). Based on these reports, a phage display antibody library for infertile patients with positive anti-sperm antibodies and for vasectomized men was created. This library was used to detect antibodies specific to sperm antigens and, among the many clones obtained, only four were strongly reactive. These four clones bear novel sequences and unique complementary sequences. FAB-7 and AFA-1 antibodies reacted with fertilization antigen 1 (Shrestha et al., 2021), whereas those for YLP20 reacted with a sperm protein that has a molecular weight of around 50 kDa. In addition, AS16 antibodies reacted with another sperm protein and caused agglutination. All these antibodies resulted in the inhibition of various sperm functions, leading to infertility. These advancements may lead to the detection of novel sperm-specific antigens for a prompt design of an immunocontraceptive vaccine.

Major detection or identification of novel antigens can be done by tracking genitally transmitted microorganisms (Sturm, 1978) from females to males during intercourse (Shi et al., 2007). This exchange may sometimes cause immune-related infertility in men (Weissenbacher et al., 2014, Beagley et al., 1998, Taylor-Robinson, 1986). Therefore, peptides derived from microorganisms identified as infertility causative agents may be grouped, after the construction of a library, and screened for unique determination sequences. Clones or proper antigens could then be derived. This method can be used as an effective alternative strategy to develop an immunocontraceptive vaccine (Shi et al., 2007).

## 4 Vaccines for sexually transmitted diseases (STDs)

Vaccine development against STDs or organisms is multiplying year by year. To develop a vaccine for an STD, the first thing is to understand the biology of the sexually transmitted organism and then the underlying mechanism (Gottlieb and Johnston, 2017, Velan and Yadgar, 2017). Unraveling the mechanism behind the infection and its transmission to the male or female partner is the most significant task in generating an excellent vaccine (Kahle et al., 2012). To produce or to make global sustainable, STI control is in need (Korenromp et al., 2016). To achieve this, vaccines against STIs are being evolved by researchers worldwide (Low et al., 2017). However, producing vaccines against STIs is difficult. Moreover, production is hindered by the fact that infections are spreading worldwide and interfere with each other. Vaccines against Hepatitis B virus (HBV) and HPV have been shown to be highly effective for prevention in humans (Kurosky et al., 2017, Van Damme et al., 2017, Gupta et al., 2017, Borch et al., 2017). This was complemented by frequent public awareness programs (Wilson, 2017, Hasan et al., 2017) regarding the use of these two vaccines. Nowadays, these two infections are under control all over the world, and guide researchers in their work for developing new STI vaccines to control other infections (Gottlieb et al., 2016). HSV-1 and 2 are the most frequently transmitted viruses via sexual interaction (Nahmias et al., 1989). An estimated population of 420 million people is globally affected or infected with HSV-2 (Looker et al., 2008) or herpes, the most common genital infection, whereas HSV-1 contributes with 100 million infected people (Stingley et al., 2000). In 2012, the World Health Organization (WHO) reported that around 400 million people in the world were infected with curable STIs; among these, *Neisseria gonorrhoeae* and *Treponema pallidum* (syphilis) were found to be prevalent. In 2013, the WHO and National Institutes of Health (NIH) decided to develop new strategies to overcome the limitations of STI vaccines. These resulted in high priority research strategies to produce vaccines against *N. gonorrhoeae* (Moncada et al., 2017); *T. pallidum* (Smolak et al., 2017), and HSV-1 and −2 by various researchers in 2014. Since then, studies have also focused on modelling the theoretical impact of STIs, analyzing cost-effectiveness of STI vaccine development, understanding the history of STIs, obtaining extensive and updated epidemiological data for various STIs, conducing translational clinical studies for STIs, encouraging both investors and pharmaceutical companies to invest in STI vaccine development with various promotion programs, and creating awareness in the public by conducing programs in rural and township areas through local public health ministries (Gottlieb et al., 2016).

## 5 Herd immunity and its effects against vaccines for STDs

In a group of populations, the acquired immunity that helps reduce the risk of spreading or adapting infections among vulnerable individuals is called herd immunity (Anderson et al., 1992). Individually, immunity can be acquired by natural infections and/or vaccination. Herd immunity can be best understood by understanding the reproductive infection number (R). R_0_ is defined as the basic reproductive number that determines the spread of an infection; it is the average number of new infections spread by one infected human being within an entirely susceptible population (Smith et al., 1984, Garnett, 2002). Acquired immunity plays a major role in the determination of this number; when acquired immunity is present in a population, such population is no longer entirely susceptible. R_t_ is the average number of very new infections caused in a susceptible population at time interval “t”. Thus, the product is said to be R_0,_ when the whole population is susceptible, but R_t_ may be proportionally smaller as the number of immune individuals within the population increases (McLean and Blower, 1995).

## 6 Herd immunity in STIS

There are two major reasons for which vaccination programs against STIs are being organized (Linares). First, the risk of transmitting and acquiring infections is increasing because of their complex heterogeneity. Second, STIs affect the population of sexually active adults and are restricted to minorities; STIs most commonly affect women than men (Hamilton and Morris, 2015). Sexually, both men and women are the reason, but the blame and the cause are usually attributed to women (Badawi et al., 2021). Thus, vaccination programs should be conducted in rural development areas to protect sexually active age groups, mainly women. Low efficacy vaccines can be used, considering that there are considerable health gains and benefits. Still, the elimination of STIs is very difficult and improved efficacy can be associated with diminishing returns in time. Herd immunity and its relatively low risk of infections have been previously explained (Garnett and Bowden, 2000). Nevertheless, in a study done on a population with different STIs, where the group with higher levels of risk was expected to transmit the infections, it was seen that most of the infections were acquired by the low-risk population instead (Garnett, 191(Supplement_1):2005). Interestingly, vaccination helped reduce the number of infections in low-risk groups and among the population with low reproductive potential (Tack et al., 2017). However, some residual infections in this population may indicate that its members had been frequently exposed to infections or were prone to the highest degree of infection (Tack et al., 2017). Such a population could potentially infect or transmit the infections to other individuals within the group. Then, control becomes assortative. Herd immunity may also help protect one sex, either male or female, through vaccinating the other.

Vaccinating individuals of a single sex against STIs may be appropriate because women, as stated before, are at a higher risk of acquiring such infections. A program like this is straightforward, as vaccination can be done at the time of family planning or antenatal care (Brabin et al., 2006). As heterosexually transmitted infections can be prevented by vaccinating only one sex, thereby indirectly protecting the other, it is a logical strategy to achieve herd immunity (Frost et al., 2014). Herd immunity helps control the infection in the directly protected sex, allowing only few exposures in the indirectly protected sex group. In the absence of vaccines for STIs, there are many practices useful for reducing their spread. First, by reducing the duration of individual infections by antimicrobial or antibiotic treatment (Dunkle et al., 2008). Second, by reducing the number of sexual partners by conducting sex education programs and informing sexually active men and women about STIs (Low and Broutet, 2017). Third, by reducing STI transmission (even when having multiple partners or sexual contacts) through the use of condoms and other protection methods (Lee et al., 2016). All these practices may be combined to reduce R_0_. Vaccines for STIs with a low degree of protection show similar effects on reducing R_0_. Suppose the practices mentioned above show a low efficiency in diminishing the infections, then, the combined effect of vaccines for STIs and other these practices may help eliminate the infections or further diminish their transmission among individuals (Rhodes et al., 2017).

## 7 Advancements in vaccine development for Syphilis

Administration of Penicillin G is currently the only treatment at all the stages of syphilis (Gottlieb et al., 2014, Ganesan et al., 2014, Xiao et al., 2017, Cao et al., 2017). The US Centers for Disease Control and Prevention (CDC) have recommended this treatment, even for pregnant women, for the past 70 years (Yang et al., 2015, Workowski, 2015, Ghanem, 2015). Although there exists an emergence of resistant *T. pallidum* strains (Manteuffel et al., 2016, Arora et al., 2017, Beale et al., 2021, Stamm, 2015). penicillin G remains as the only effective treatment against syphilis. Therefore, *T*. *pallidum* sometimes persists after penicillin treatment. Thus, to eradicate this disease, we have been relying on public education programs about syphilis and its pathogenic nature, such as that organized by the CDC in 1999 and the National plan to eradicate syphilis, held in 2006 (Stamm, 2015, Smajs et al., 2015, Wu et al., 2016), but the disease is still spreading. The WHO has also taken initiatives for the global eradication of syphilis, especially congenital syphilis (*i.e.*, the transmission of the infection from the mother to the fetus). This last program was a complete success; the first country to achieve this goal was Cuba (Ellington, 2016). However, as per the current situation, neither treatment nor awareness education programs are reducing syphilis transmission, and there is an urgent need for the development of a vaccine against this disease (World Health Organization, 2017, Cameron and Lukehart, 2014, Lithgow and Cameron, 2017, Champredon et al., 2016). An effective vaccine for syphilis should prevent *T*. *pallidum* infections, thus preventing disease transmission among sexual partners and between infected mothers to their children; it should also provide protection from diverging *T*. *pallidum* strains and decrease reinfection frequency. By eliminating reinfection, penicillin may endure as an effective syphilis control method (Garnett, 2014). All this information and requirements should be helpful for designing an effective vaccine for syphilis, in our way to eradicate this infection.

Compared to other pathogens, only a limited number of studies exist on the development of a vaccine for syphilis (Peterman; T.A. and B.W. Furness, 2015). This is because only a few scientists are working on this area and because the pathogenesis of *T. pallidum* and its morphological structure are still not completely understood. In addition, there are no studies about the genetic modifications among *T. pallidum* that could be important for vaccine development (Tong et al., 2017).

Metzger did the first vaccination studies for syphilis. *T. pallidum* strains were attenuated, by storing them at 4 °C for a short time (Zuckerman and Harper, 2016), and used to inoculate rabbits by the intramuscular route. This gave good results, and further lesions in the rabbits were not noticed. This historical investigation results led Miller to work on it further by inoculating rabbits with bacteria attenuated by γ-irradiation (Metzger and Smogor, 1969). This method resulted in a labile surface antigen content in rabbits and thus in an attenuated infection. From this study, Miller concluded (a) that the development of protective immunity was based on the presence of intact bacteria and that the bacterial surface should not be disturbed during attenuation (Miller, 1973); (b) that prolonged immunization was necessary to get good results given that the number of proteins in the outer membrane of *T. pallidum* is smaller than that in other bacteria; and (c) that achieved protection was long-lasting. The study also concluded that the rabbit model was suitable for this kind of studies.

*T*. *pallidum* can develop chancres (Hoffman et al., 2002) at the site of infection, resulting in repeated reinfections and the establishment of latency despite the immune response produced by the vaccine. Therefore, this phenomenon should be considered a key element in designing a successful vaccine for syphilis. An effective vaccine should prevent chancre development and treponemal persistence and should avoid reinfection (Hoffman et al., 2002). This could help eliminate the disease symptoms in infected individuals and prevent its transmission in the population. Key considerations for developing a successful vaccine for syphilis at the pre-clinical level are the number of vaccine doses needed for obtaining the best immunity, vaccination administration methods, duration of post immunity, optimization of a multivalent vaccine, and adjuvant selection (Fukuda et al., 2015).

There has also been some advancements in syphilis pre-clinical trials using quantitative real-time PCR (RT-qPCR) (Dodet, 2014). This allows us to find and detect the sensitive *T*. *pallidum* DNA at distal sites in the rabbit. However, there are some limitations with RT-qPCR. This led to the use of fluorescence *in situ* hybridization (FISH) to detect *T*. *Pallidum* in human and animal model infected tissues (Kotloff).

This also led to further investigations for the design of an effective vaccine for syphilis. However, the development of such vaccine was understudied as only a specific group of scientists were working towards *T. pallidum* pathogenesis. Further, conducting many awareness programs about *T. pallidum* in particular geographical locations where the infection and transmission rates are high may improve sexual habits among the population, providing better results (Manteuffel et al., 2016, Arora et al., 2017). Therefore, government and private funding agencies should focus in investing in projects for developing a new vaccine for syphilis for the betterment of humankind. In addition, developing a vaccine for both syphilis and any another STI may yield good profits and broaden the market for the syphilis vaccine (Petrich et al., 2015). In conclusion, an effective vaccine for syphilis will protect humankind from infectious and congenital syphilis.

## 8 Advancements in vaccine development against *Neisseria gonorrhoeae* (Gonococcus)

Gonococcus is considered as one of the most dangerous human pathogens since ancient days. Gonococcal infections in humans are transmitted through direct contact (Best and Pa, 2015, Lister et al., 2003). The major transmission routes involve sexual contact, by touching mucosal membranes of the male and female genitals (Rice et al., 2017), through the anal canal (either between heterosexual (Golden et al., 2005) or homosexual partners (Kent et al., 2003), or though the oropharynx (Fingerhuth et al., 2016, Bernstein et al., 2017, Yang et al., Sextrans-2017., Zhang et al., 2017, Zhang et al., 2017, Lewis, 2015, Chow et al., 2016); it may even be transmitted, rarely, through eye contact (Nakashima et al., 2014, Kreisel et al., 2017). Except for that involving eye contact, any other mode of transmission may be restricted by conducting multiple education programs to the population at reproductive age. Such sex education programs, including safety measures for those with multiple sex partners and/or homosexual habits, might work at the highest level for eradicating gonococcal infections throughout the world, especially in the western countries (De Silva et al., 2016, Sarkar et al., 2016, Huneeus et al., 2018, Eldredge et al., 2016, Pelligrino et al., 2017, Yeung et al., 2017). Gonococcal infections lead to cervicitis (Gonzalez et al., 2017, Lama and Kayastha, 2017, Gorgos et al., 2017, Igietseme et al., 2015) in women and urethritis (Bautista et al., 2016, Horner et al., 2016, Priest et al., 2017, Bachmann et al., 2015) in men, but they may be cured if medical treatment is immediately sought. Mainly, asymptomatic men are responsible for the high transmission rates of the disease through sexual contact (Dudhipala et al., 2017, Ong et al., 2018), therefore, finding the correct symptoms for gonococcus infection is necessary for restricting the transmission (Pattanasin et al., 2017). Although most women are found asymptomatic, if untreated, there is a chance for developing pelvic inflammatory disease, especially salpingitis (Handsfield, 1972), and even infertility (Fein et al., 2015). There is also a chance of ectopic pregnancy, which is highly painful for women. In some untreated men and women, gonococcus might enter the bloodstream and cause meningitis.

The CDC officially announced that gonorrhea is the second most threating bacterial infection in the world, with the highest transmission rates (Lister et al., 2003). Importantly, there was a 27% increase in this transmission rate among individuals in the reproductive age group from 2012 to 2015. Currently, antibiotic therapy is the only way to cure the disease and stop the transmission between individuals (Briceag et al., 2015). However, recently, *N. gonorrhoeae* strains have become resistant to many commercial antibiotics, those extensively used to cure gonorrhea (Balashov et al., 2016). Consequently, there is a need for vaccines or antimicrobial peptide treatments to deal with these resistant strains. Third-generation cephalosporins are the antibiotics that have been used from the 1980s to present days for the treatment of gonorrhea (Tuite et al., 2017), whereas sulfonamides were used from early 1938 to 1942 (Regnath et al., 2016). It is now known that single nucleotide polymorphisms in the *folP* gene reduce the affinity of the resulting protein for sulfonamides, leading to bacterial resistance. Historically, for more than four decades, penicillin was the only ruler against gonorrhea, whereas tetracycline, doxycycline, and spectinomycin were also used in the treatment of gonorrhea from the 1970s to 1980s (Rodriguez).

The search for a vaccine for gonorrhea dates back to the early 20th century (Washington, 1979), specifically to the 1910s, but it was not successful. However, as many antibiotics were effective for curing gonorrhea, a vaccine was not really necessary. At least, this was the mentality of the researchers at that time. However, four candidate vaccines were tested afterwards, but none of them protected individuals from acquiring the infection. Vaccine development for gonorrhea was stopped for nearly 10 or 15 years for several reasons. With the emergence of multi-drug resistance strains throughout the world, the search for a gonorrhea vaccine was reactivated in early 2010 in a full-time way (Abbasi, 2017).

A surprise came for the researchers at New Zealand in the form of a meningococcal vaccine that also showed promising results against gonorrhea. It was noticed that MeNZB (Petousis-Harris et al., 2017) was 31% effective in preventing gonorrhea. Though MeNZB is no longer produced, its active ingredients are present in 4CMenB. This vaccine (Lennon et al., 2012) contains three genetically modified proteins and two proteins shared between *N. meningitidis* and *N. gonorrhoeae*. 4CMenB was found to be a very effective vaccine for gonorrhea in double-blind studies and in placebo-controlled trials, with approximately 31% protection against the disease. However, it is believed that it is even more effective as an 80 million cases reduction in gonorrheal infections was registered since its production began.

The development of effective vaccine candidates for gonococcal infections is difficult because the human body's immunological protection is changing and not predictable. There is also a lack of scientists vigorously working with gonococcal infections and vaccine development (Santolaya et al., 2012). Surface antigenicity for gonococcus is modifiable and utilizes phase variation (Trembizki et al., 2015). This capacity has allowed gonococcal bacteria to adapt to any environment, and many changes have occurred since their origin (Palmer, 2016). An effective vaccine should overcome or bypass these adaptations, which should also be of concern for the development of new vaccines. Adaptive immune mechanisms of gonococcal antigens during infection do not eliciting a long-lasting protective immune response, therefore, there is a chance for reinfection.

Two vaccines against gonococcal infections were designed. One of them was based in whole-cell bacteria and the other included a single antigen. Both yielded attention to the developers, but they practically failed in giving any protection against gonococcal infections (Zielke et al., 2016). Vaccines for *N. gonorrhoeae* using bacterial antibody activity directed towards gonococcal infections are being assessed. Theoretically, bacterial antibodies would directly kill gonococci and promote phagocyte binding through complement receptors and the designed FC, helping to eliminate the infecting organisms (Lam, 2017, Edwards et al., 2016, Piekarowicz et al., 2016, Lao and Bonavida, 2016, Butler et al., 2018). The most frequently used antibodies are Por and LOS, which bind to their antigenic targets on gonococci outer membrane through complement receptors. Many ideas and knowledge can be shared at the theoretical level, but gonococci have demolished practically all this scientific knowledge towards effective vaccine development in numerous ways. Nevertheless, researchers are still working with gonococci surface antigens or complement receptors that elicit antibodies and that might be excellent targets and vaccine candidates (Gulati et al., 2015, Semchenko et al., 2017, Shewell et al., 2017). Interestingly, some important antigenic sites that would not elicit any immune response or antibodies in natural infection, might also be potential targets for vaccine development (Gunderson and Seifert, 2015, Rotman and Seifert, 2015). Transcriptome analyses from the genital tract of infected men and women will pave the way for effective vaccine design.

There is an urgent need for a gonorrhea vaccine owing to the rapidly developing multi-drug resistant *N. gonorrhoeae* strains*.* These changes occur through various mechanisms, such as plasmid acquisition, point mutation, and naked DNA uptake from other gonococcal strains. Currently, there is no reliable antibiotic or empirical treatment for this disease as well (Zhang et al., 2016). The absence of robust animal models for mimicking the prototypic disease and its variants also challenges immune response predictions (Vincent and Jerse, 2018). Therefore, new discoveries in the horizon of vaccine development are a welcome step. First among them is the finding that *N. gonorrhoeae*, for its benefit, manipulates the host system. Second, new developments in an animal model for pre-clinical evaluation of gonococcal vaccines are needed. Third, to tap conserved surface antigens of *N. gonorrhoeae* for vaccine development. Recently, vaccines, such as MeNZB and Bexsero, have been developed for gonorrhea (Edwards et al., 2018).

Gonorrhea not only threatens the general public but also military personnel. Thus, the US military has strongly recommended vaccine research and development (Russell, 2018). *In vitro* and clinical studies, proteomics-driven antigen discovery, and comprehensive bioinformatics strategies will help make informed and rational decisions on vaccine development. Pertaining to gonorrhea, structural vaccinology is in its infancy and the response should be public alarm; consequently, research on vaccine development for gonorrhea is urgently needed (Ratto-Kim et al., 2018).

## 9 Current status of STD vaccines

The current status of the vaccines for STDs and their administration is tremendously different depending on each region and its demographic characteristics. It is mostly influenced by the awareness of the disease and the cultural acceptability of vaccines in every specific area. Most of the vaccines associated with STDs still have to pass some key checkpoints before landing up into the market space. The successful achievement of endpoints in clinical trials and the validation of surrogate endpoints is crucial. Regulatory routes need to be determined well for licensing. Systems to monitor vaccine responses in advance are always appreciated and sought after in great demand.

## 10 HPV vaccine

A bivalent form of vaccination is actively used against HPV16 and 18; it is commercially known as Cervarix, and it was approved in 2007. Gardasil 9 was approved in 2016; it is actively administered against HPV6, 18, 11, and 16. This nonavalent vaccine is the only one offered in the United States. The dosage has been reduced to 2 from 3 (Dobson et al., 2013). For women nearly 45, three doses are recommended, followed by immunosuppressants. The latter may be given to any person, regardless of gender and age. The prevalence and distribution of HPV types differ by geographic region (Stanley et al., 2012). All three prophylactic combination vaccines contain L1 in its purified form and recombinant HPV empty shells (Stanley et al., 2012). The levels of vaccine content in the serum of the patients varies with age with maximum seropositivity seen for ages between 15 and 25 years. Serum levels reduce a bit as patients grow older (Dadar et al., 2018). Even a vaccine-based therapy is available for HPV, which triggers the cell-mediated immunity. The nonavalent prophylactic vaccine is the best vaccine for HPV in the present-day scenario (Anna Rosa Garbuglia).

## 11 *N. Meningitidis* & Syphilis vaccine

Group B OMV (outer membrane vesicle) vaccines against *Neisseria gonorrhoeae* have been developed. But the pathway and mode of action are still very unclear. Hurdles like the relation between pelvic inflammation and infection, biomarker development, and specific tests for upper genital tract ailments are seen during clinical trials. The male urethral model is also a serious challenge that is still stuck at clinical trials phase II. In the upcoming years, the effect of 4CMenB against gonorrhea deserves special attention (Registry, 2020). The first prototype vaccine for syphilis was designed in 1973 (Miller, 1973). Animal models administered with *T. pallidum* treated with γ radiation showed protection against the disease for 1 year (Cameron; C.E. and S.A. Lukehart, 2014). Subsequently, *Borrelia burgdorferi* based carrier and *T. pallidum* flagellin encoding plasmid were proposed as candidate vaccines, but these are still in experimental stages (Parveen et al., 2019, Zheng et al., 2018). Due to ethical reasons and safety concerns of the subjects under clinical trials, the vaccine for syphilis has not completed human trials yet (Centre for Disease Control and Prevention. The Tuskegee timeline, https://www.cdc.gov/tuskegee/ timeline.htm. (accessed 13 August 2019).

## 12 *Chlamydia* vaccine

Modelling studies have suggested that partial protection of *C. trachomatis* (Ct) could be a cost-effective vaccine. But the burden of treating Ct-associated diseases, especially in developing and underdeveloped countries, makes potential vaccination inevitably necessary. Trials are still being completed in humans for Ct vaccines as endpoints for the efficacy are still under research. Scientists are currently working on biomarker identification associated with inflammation for upper genital tract and the role of antibodies (CD4 T cells generated by Ct specific vaccine). There are several potential vaccines available today for Ct which are administered in combination by practitioners; they include NanoBio Corp’s Intranasal MOMP nanoemulsion with Fattom, Statens Serum Institut’s MOMP-VD4 neutralizing antibodies, Pan-Provincial Vaccine Enterprise Inc. and British Columbia CDC’s MOMP + Pmps, Selecta Biosciences’s cSAP TLR7 agonist with UV-killed Chlamydia, Prokarium’s Vaxonella platform, and NIH/NIAID plasmid-deficient trachoma vaccine (Olsen et al., 2015, Bøje et al., 2016, Olsen et al., 2017, Fattom, 2019, Karunakaran et al., 2015, Stary et al., 2015, Garmory et al., 2005, Kari et al., 2011).

## 13 HSV vaccine & HIV vaccine

Preclinical forms for HSV treatment and prevention are mutated forms of HSV-1, HSV-2, glycoprotein B lentiviral vector with HSV-1, MPL/alum in HSV-2, intranasal recombinant of HSV-1 g, and trivalent glycoprotein. Although mice and guinea pig models are not reliable for human responses for this specific disease, these models have reported enhanced viral clearance and reduction recurrence of the ailment due to the vaccine (Corey et al., 1999, Mascola, 1999, Strasser et al., 2000). The vaccines that are underway for simplex virus associated with humans are Profectus BioSciences, gD2/ICP4, GEN-003 Genocea, HerpV Agenus, HSP, QS-21 with 32 to 35-mer peptides and polynucleotides, gD2+/–UL46 Vaxfectin, and HSV529 Sanofi Replicated attenuated HSV-2X (Johnston et al., 2016). These are generally therapeutic vaccines that might surface in the coming years as all of them have reached phase II of clinical trials except for HSV529 Sanofi Replicated attenuated HSV-2X. NIAID of the NIH, US Army Medical Research and Development Command, Janssen, and the HIV Vaccine Trials Network has been working on an acceptable vaccine model which addresses the genetic diversity of HIV (ClinicalTrials.gov. Identifier NCT0, 2019). The steps that need to be rendered in the subsequent years should be the completion of clinical trials of mosaic and prime boost approach vaccines. Few similar therapies like vaccines available today for HIV are immunogen stimulating germlines, passive immunization antibodies, envelope domains, envelope trimers, and genetic delivery by viral vectors and replicating viral vectors (Rerks-Ngarm et al., 2009, Jardine et al., 2013, Moldt et al., 2012, Lewis et al., 2002, Parks et al., 2013).

## 14 Zika virus vaccine

In the early months of 2020, zika virus was found to be dormant but the cohort presence of the viral strain in infants infected between 2015 and 2017 is still a medical issue that needs addressing (Staples et al., 2020). The viral RNA has been found to be prevalent in the semen of symptomatic patients for up to 6 months (Counotte et al., 2018). The WHO is currently working on a set of vaccines for tackling zika virus, to name a few, AGS-v with salivary proteins, mRNA-1325, ZIKV, PIZV or TAK-426, VRC-ZKADNA085-00-VP, GLS-5700 DNA prME, and MV-Zika (World Health Organization, 2019, Sakkas et al., 2018).

## 15 Conclusion and future perspectives

Integrative approaches with advancements in proteomics, immunomics, and immunogenomics will help in the development of a well-suited contraceptive vaccine for the next millennium. There is an imperative need to control the population growth rate throughout the world, utilizing innovation in contraception. In developing countries, between the first and second pregnancies, women undergo frequent abortions due to a lack of knowledge about contraception, affordability, and even failure when using existing contraceptive measures. Besides, contraceptive measures should be one of the main focuses of the next millennium, as 46 million abortions are being done yearly in developed nations. To our knowledge, contraceptive vaccines could be the choice to overcome the drawbacks in the existing contraception, *e.g.*, those related to condom usage. Fortunately, many contraceptive vaccines are being formulated, and some have been successful in animal models and are soon expected to undergo clinical trials. But still, we hope that advancements in integrative approaches with multi-omics techniques may come a practice to humankind in the near future. Before the development of vaccines for STIs, to understand the mechanism of transmission of organisms between individuals and the nature of STIs causing organisms is a must. If the transmission is clear, the vaccine development against each STI becomes easy and straightforward. Even though we have vaccines for hepatitis B, and HPV, the prevalence of these infections is still at an alarming stage. This is because of a lack of knowledge about STIs in rural areas and because government agencies are not conducting frequent awareness programs about STI vaccines. A lot of groundwork is in need for implementing the vaccines against STIs for prevention and control. Hopefully, vaccines for contraception and vaccines against STIs will be available in the market for the betterment of humankind in the near future. In a clinical context, a safe contraception vaccine, with no side effects, and reversible is crucial; however, it is also difficult to develop because human immune responses after immunization are hard to understand and sometimes inevitable.

## Author contributions

All the authors substantially contributed to the conception, compilation of data, checking and approving the final version of the manuscript, and agree to be accountable for its contents.

## Declaration of Competing Interest

The authors declare that they have no known competing financial interests or personal relationships that could have appeared to influence the work reported in this paper.
